# Insights into the flexibility of the domain‐linking loop in actinobacterial coproheme decarboxylase through structures and molecular dynamics simulations

**DOI:** 10.1002/pro.70027

**Published:** 2025-01-25

**Authors:** Gaurav Patil, Diego Javier Alonso de Armiño, Yirui Guo, Paul G. Furtmüller, Dominika Borek, Dario A. Estrin, Stefan Hofbauer

**Affiliations:** ^1^ Department of Chemistry, Institute of Biochemistry BOKU University Vienna Austria; ^2^ Instituto de Química, Física de los Materiales, Medio Ambiente y Energía (INQUIMAE) CONICET‐Universidad de Buenos Aires Buenos Aires Argentina; ^3^ Ligo Analytics Dallas Texas USA; ^4^ Department of Biophysics The University of Texas Southwestern Medical Center Dallas Texas USA; ^5^ Department of Biochemistry The University of Texas Southwestern Medical Center Dallas Texas USA

**Keywords:** cryo‐EM, heme biosynthesis, molecular dynamics simulations, structural biology

## Abstract

Prokaryotic heme biosynthesis in Gram‐positive bacteria follows the coproporphyrin‐dependent heme biosynthesis pathway. The last step in this pathway is catalyzed by the enzyme coproheme decarboxylase, which oxidatively transforms two propionate groups into vinyl groups yielding heme *b*. The catalytic reaction cycle of coproheme decarboxylases exhibits four different states: the apo‐form, the substrate (coproheme)‐bound form, a transient three‐propionate intermediate form (monovinyl, monopropionate deuteroheme; MMD), and the product (heme *b*)‐bound form. In this study, we used cryogenic electron microscopy single‐particle reconstruction (cryo‐EM SPR) to characterize structurally the apo and heme *b*‐bound forms of actinobacterial coproheme decarboxylase from *Corynebacterium diphtheriae*. The flexible loop that connects the N‐terminal and the C‐terminal ferredoxin domains of coproheme decarboxylases plays an important role in interactions between the enzyme and porphyrin molecule. To understand the role of this flexible loop, we performed molecular dynamics simulations on the apo and heme *b* coproheme decarboxylase from *Corynebacterium diphtheriae*. Our results are discussed in the context of the published structural information on coproheme‐bound and MMD‐bound coproheme decarboxylase and with respect to the reaction mechanism. Having structural information of all four enzymatically relevant states helps in understanding structural restraints with a functional impact.

## 1 INTRODUCTION

Coproheme decarboxylases (ChdCs) are enzymes produced by Gram‐positive bacteria that are involved in the coproporphyrin‐dependent pathway (CPD) of heme biosynthesis (Dailey et al. 2010; Dailey et al. 2017; Layer 2021; Pfanzagl et al. 2018; Zamarreño Beas et al. 2022). All ChdCs are homopentameric proteins consisting of protomers of 250–300 amino acid residues, each with two ferredoxin‐like domains, with only the C‐terminal domain having a functional coproheme/heme *b* binding site (Falb et al. 2023).

The enzyme catalyzes the final two‐step decarboxylation of two propionate groups in the substrate iron coproporphyrin III (coproheme) to convert it into the final product heme *b* (Celis et al. 2017; Dailey et al. 2015; Lobo et al. 2015). Two equivalents of hydrogen peroxide are used to perform both the decarboxylation reactions (Hofbauer et al. 2016). This process of sequential decarboxylation involves the formation of a three‐propionate intermediate 2‐monovinyl, 4‐monopropionate, deuteroheme (MMD) which undergoes 90° rotation in the active site to facilitate the second decarboxylation resulting in heme *b* (Michlits et al. 2020; Milazzo et al. 2019a). A tyrosine residue was identified as the catalytically relevant radical site essential for both decarboxylation reactions in ChdCs from the firmicutes *Staphylococcus aureus*, *Listeria monocytogenes*, and actinobacterial *Corynebacterium diphtheriae* (Michlits et al. 2020; Streit et al. 2018).

The active site of *Corynebacterium diphtheriae* coproheme decarboxylase (*Cd*ChdC) has been structurally characterized and residues involved in substrate binding and catalytic mechanism have been identified (Hofbauer et al. 2021; Michlits et al. 2020; Patil et al. 2023; Sebastiani et al. 2021). Two crystallographic structures of coproheme‐bound *Cd*ChdC (6XUC) and MMD‐bound *Cd*ChdC (6XUB) are deposited in PDB.

While ChdCs from firmicutes and actinobacteria share structural features, the loop region between the N‐terminal and C‐terminal domains varies between species. Sequence alignments, homology modeling, and available crystallographic models show the variable length of the loops and their different architecture close to the active site (Celis et al. 2017; Hofbauer et al. 2015; Milazzo et al. 2019a; Pfanzagl et al. 2018). Moreover, the loop in actinobacterial structures shows a conserved histidine H118, located close to propionate at position 7 (p7) of the porphyrin ring establishing a direct H‐bond (Michlits et al. 2020). This histidine acts as a distal base and is involved in deprotonation of hydrogen peroxide leading to higher catalytic efficiency of actinobacterial ChdC as compared to ChdCs from firmicutes (Michlits et al. 2020; Sebastiani et al. 2021).

All proteins are dynamic. For example, in X‐ray crystal structures of *Listeria monocytogenes* ChdC, only two out of five subunits showed resolved loop regions, and the loops in these two subunits are stabilized by crystal packing interactions (Hofbauer et al. 2021; Milazzo et al. 2018). Conformational dynamics are often required to achieve specific functions or mechanisms, which might involve subtle side‐chain motions, larger loop jiggling, and even larger conformational changes engaging the entire domain. In this study, we analyze the loop movement of *Cd*ChdC by comparing the structural information of all enzymatically relevant states: empty (apo‐ChdC), substrate‐bound (coproheme‐ChdC), intermediate (MMD‐ChdC), and product‐bound (heme *b*‐ChdC), and present molecular dynamics simulation studies probing the loop behavior. Comparison of high‐resolution X‐ray crystallography and cryo‐EM structures provides additional information on conformational heterogeneity, in particular for protein side chains and other flexible regions (Wang and Wang 2017).

The requirement for a crystalline sample is one of the most significant limitations of X‐ray crystallography. Especially for protein samples containing regions with high conformational flexibility, obtaining crystals can be challenging. The presence of a flexible loop in the apo‐structure of *Cd*ChdC makes it quite difficult to crystallize in comparison to the coproheme‐bound *Cd*ChdC structure, in which the loop is stabilized by interactions with the substrate present in the active site (Sebastiani et al. 2023). To overcome these limitations of X‐ray crystallography, we used cryo‐EM SPR to obtain three‐dimensional structures of apo‐*Cd*ChdC (8QWC) and heme *b*‐*Cd*ChdC protein (8QUO).

## 2 METHODS

### 2.1 Protein expression and purification

Recombinant *Cd*ChdC wild‐type apo protein was expressed and purified as described previously (Michlits et al. 2020). For cryo‐EM experiments, the His‐tagged protein was cleaved with HRV 3C protease, and half of the apo‐enzyme purified by Ni^2+^ column was reconstituted with hemin, to obtain heme *b*‐*Cd*ChdC, prior to size exclusion chromatography (SEC). The SEC purified protein was concentrated and used directly for grid preparations.

### 2.2 Cryo‐EM grid preparation and data collection

The SEC purified samples of apo*Cd*ChdC and coproheme‐*Cd*ChdC were applied to Quantifoil R1.2/1.3 300 Mesh Gold and Copper grids, respectively. The grids were subjected to pretreatment with glow discharge for 90 s at 30 mA by PELCO easiGlow Glow Discharge cleaning system to remove the adsorbed hydrocarbons and to generate hydrophilic surface. The grids were placed inside the Thermo Scientific Vitrobot Mark IV System to prepare vitrified samples by the process of plunge freezing. Three microliter of purified protein samples with concentrations varying from 70 to 120 μM were applied to the glow‐discharged surface of the grid at 4°C at 100% humidity. A blotting force of either 18 or 19 was applied for 5.0–5.5 s to remove the excess solvent from the grid surface. The grids were then plunged frozen into liquid ethane, clipped on a clipping station, and stored in liquid nitrogen until data collection.

The data were acquired with a G2 300 kV FEI Titan Krios microscope (Thermo Fisher) equipped with a Gatan K3 direct electron detector (6 K × 4 K) run in super‐resolution mode at a nominal magnification of 105,000×, with a physical pixel size of 0.834 Å. SerialEM was used for automated data collection with multi‐hole targeting using a beam‐image shift approach to collect multiple images per stage movement (Mastronarde 2005). An optimal defocus range of −1000 to −3000 nm was used to generate phase contrast. The slit width of the GIF Quantum Energy Filter was set to 25 eV. Movies were dose‐fractionated into 112 frames with a total electron exposure of 72 e^−^/Å^2^. The total number of movies collected for apo*‐Cd*ChdC and heme *b‐Cd*ChdC were 1035 and 1,300, respectively.

### 2.3 Cryo‐EM image processing and model building

Cryo‐EM Single‐Particle Ab Initio Reconstruction and Classification (CryoSPARC) software was used for processing the single‐particle cryo‐EM data of apo‐*Cd*ChdC and heme *b‐Cd*ChdC protein. All movies were imported in .tiff file format followed by patch motion correction and CTF correction (Punjani et al. 2017). Particle picking was performed using a blob picker with setting a minimum particle diameter of 100 Å and a maximum diameter of 220 Å. After inspecting the picks for false‐positive particles, the particles were extracted with the box size of 432 to generate initial 2D templates. The selected 2D classes' templates were applied to all the micrographs using a template picker and extracted using a box size of 432 for apo‐*Cd*ChdC and 352 for heme *b‐Cd*ChdC protein. This was followed by several rounds of 2D classification that resulted in a final 638,556 particles of apo protein and 870,660 particles of heme *b*‐bound protein. Ab initio reconstruction on the clean stack of particles and one round of homogenous refinement with C5 symmetry was conducted which resulted in density maps of 2.27 Å for apo and 1.94 Å for heme *b*‐bound protein.

MOLREP software implemented in CCPEM was used to perform molecular replacement by docking PDB model 6XUC against the generated density map of apo‐*Cd*ChdC and heme *b‐Cd*ChdC protein (Burnley et al. 2017; Vagin and Teplyakov 1997; Wood et al. 2015). The model was then iteratively rebuilt in Coot and refined using Phenix.real_space_refine and Refmac_Servalcat (Afonine et al. 2018; Brown et al. 2015; Krissinel and Henrick 2007; Liebschner et al. 2019; Murshudov et al. 2011; Yamashita et al. 2021). Model validation and summary statistics were generated by MolProbity in Phenix as well as model validation tool in CCPEM (Chen et al. 2010) (Table 1).

**TABLE 1:** Data collection and refinement statistics.

Data collection		
Instrument	Titan Krios G2	
Detector	K3 Gatan (6 K × 4 K)	
Energy filter	Yes	
Nominal magnification	105,000×	
Data collection mode	Beam‐image shift; 3 × 3 per stage position (multi‐shot targeting, 4 shots per hole)	
Frames per movie	112	
Total electron dose (e^−^/Å^2^)	72	
Exposure time (s/frame)	0.0407	
Detector pixel size (Å)	0.834	
Data pixel size (Å)	0.417	
Movies acquired	1,035 (apo‐*Cd*ChdC)	1,300 (heme *b*‐*Cd*ChdC)
Reconstruction		
	apo‐*Cd*ChdC (8QWC)	heme *b‐Cd*ChdC (8QUO)
Measured molecular weight (kDa)	136.3	136.3
CTF correction method	Phase flipping and amplitude correction	Phase flipping and amplitude correction
Reconstruction symmetry	C5	C5
Particles used in refinement	638,556	870,660
Resolution FSC_0.143_ (Å)	2.27	1.94
Refinement and validation		
	apo‐*Cd*ChdC	heme *b‐Cd*ChdC
Non‐hydrogen atoms	9,340	11,144
Protein residues	1,100	1,150
Ligands	0	5
RMSD bond lengths (Å)	0.004	0.003
RMSD bond angles (°)	0.525	0.544
Model‐to‐map FSC (all)	0.90	0.92
MolProbity score	1.17	1.24
Clashscore (all atom)	3.82	4.9
Poor rotamers (%)	0.87	0.71
Ramachandran (%)		
Favored	99.72	98.51
Allowed	0.28	1.49
Outliers	0	0

### 2.4 Molecular dynamics of the loop in the apo and heme *b*‐bound structures

Molecular dynamics simulations were performed for the apo‐*Cd*ChdC and heme *b*‐bound proteins. The study aimed to determine the movement of the loop close to the active site and its role in orienting and stabilizing the substrate within the active site cavity.

Initial structures were obtained from Protein Data Bank (PDB code 6XUB) (Michlits et al. 2020). The full pentameric form was used for the simulations. The heme‐*b* charges and bonding parameters were the same as previously published (Capece et al. 2008; Capece et al. 2013; Nadra et al. 2008), which have been extensively tested and validated in conjunction with the Amber force field ff99SB. All MDs were performed using the Amber 20 molecular dynamics simulations package (Salomon‐Ferrer et al. 2013). Solvated initial structure was generated using tleap program adding TIP3P‐type water molecules up to a radius of 10 Å truncated octahedral box. 25,000 steps of steepest descent energy optimization followed by another 25,000 of conjugate gradient energy optimization were performed to prevent close contacts. After running 60,000 steps of NVT simulation with a 0.5 femtosecond (fs) timestep and a linear ramp of the Langevin thermostat from 10 to 100 K, harmonic restraints were applied to all protein atoms using a spring constant of 1.0 kcal mol^−1^ Å^−2^. This was followed by a second NPT thermalization round using a 1 fs timestep and a temperature ramp from 100 to 298 K. After heating, six rounds of equilibration were performed in the NPT ensemble at 298 K and 1 atm with decreasing restraints of 0.8, 0.6, 0.4, 0.2, 0.1, and 0.05 kcal mol^−1^ Å^−2^, respectively, where the restraints were applied only to backbone atoms in the last four rounds of equilibration. Production trajectories were obtained in the NPT ensemble using the Berendsen barostat (Berendsen et al. 1984) and Langevin thermostat (Brooks 1989), a timestep of 2 fs, and a final temperature of 298 K with no additional restraints of any kind. A cutoff for non‐bonding interactions of 12 Å and the SHAKE algorithm for constraining light atom bond distances were applied in all the MD simulations.

For the apo‐protein, a total of 2 μs of NPT (constant number of particles, pressure of 1 atm, and constant temperature of 298 K) MD simulations in three replicas were obtained (1 × 800 ns, 2 × 600 ns replicas). For the heme *b*‐*Cd*ChdC complex, a total of 3.72 μs of NPT‐MD simulation was divided into four replicas of 0.88–1.0 μs. All simulations were performed with explicit solvent (TIP3P water) in truncated octahedral boxes with periodic boundary conditions.

For clusterization analysis, an agglomerative hierarchical clustering (AHC) algorithm was employed using RMS as the distance metric over the loop region. The structural flexibility of these clusters was measured using the root‐mean‐square fluctuation (RMSF) parameter, defined as the residual fluctuation of every single atom about its average position. The CPPTRAJ program of the AmberTools 23 package was used for the analyses (Case et al. 2024).

## 3 RESULTS

### 3.1 Cryo‐EM structure analysis of apo‐ *Cd*ChdC and heme *b‐* *Cd*ChdC

**FIGURE 1:**
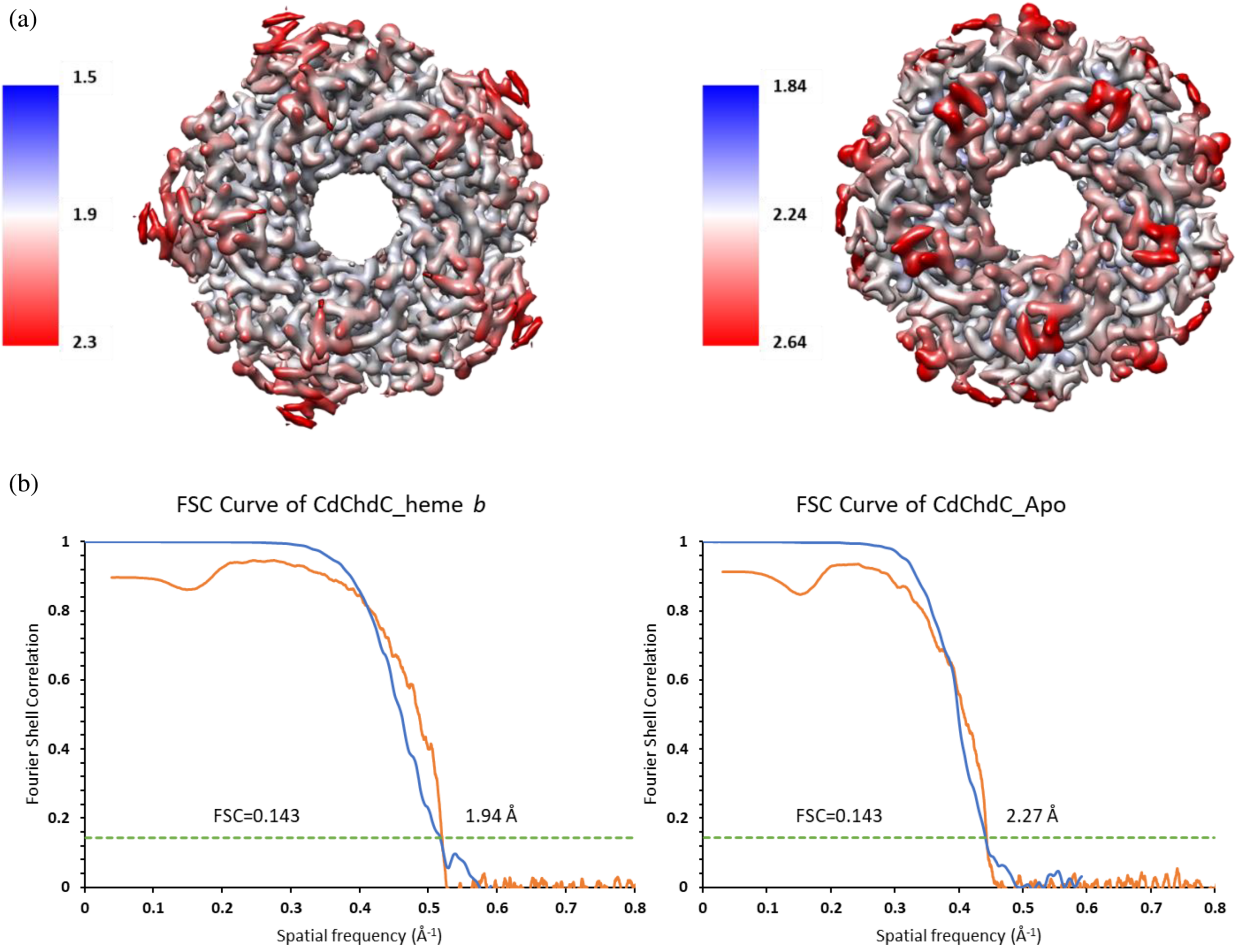
(a) Cryo‐EM density map of the structure of heme *b*‐*Cd*ChdC (left) and apo (right) pentamer, colored by local resolution estimation obtained with cryoSPARC and ChimeraX. (b) Gold‐standard Fourier shell correlation (FSC) curves for the cryo‐EM SPR structures. FSC cutoff value of 0.143 results in spatial frequency of 1.94 Å and 2.27 Å for (a) heme *b*‐CdChdC (blue) and (b) apo‐*Cd*ChdC (blue), respectively. FSC between the experimental maps and the models are represented in orange.

Attempts to solve the apo and heme *b*‐bound structure of *Cd*ChdC failed with X‐ray crystallography. While structures of apo‐ChdCs from solely non‐actinobacterial representatives (PDB ID: 4WWS, 1T0T, 1VDH, 6SVA, 6SVC) were successfully solved by X‐ray crystallography or cryo‐EM, no heme *b‐*bound structure of any ChdC has been reported until now (Bromberg et al. 2020; Ebihara et al. 2005; Hofbauer et al. 2015). In this work, the cryo‐EM structures of apo‐*Cd*ChdC (8QWC) and heme *b‐*CdChdC (8QUO) states were solved at a resolution of 2.27 and 1.94 Å, respectively (Figure 1).

In the cryo‐EM structure of heme *b‐Cd*ChdC, amino acid residues N6‐G235 were observed, while in apo‐*Cd*ChdC N11–P111 and S117–G235 residues were observed but there was no density for the N‐terminal residues as well as A112–R116 due to disorder. The local resolutions maps indicate that the N‐termini and C‐termini and the loop connecting the two ferredoxin‐like folds have lower resolution than the rest of the structures (Figure 1a).

**FIGURE 2:**
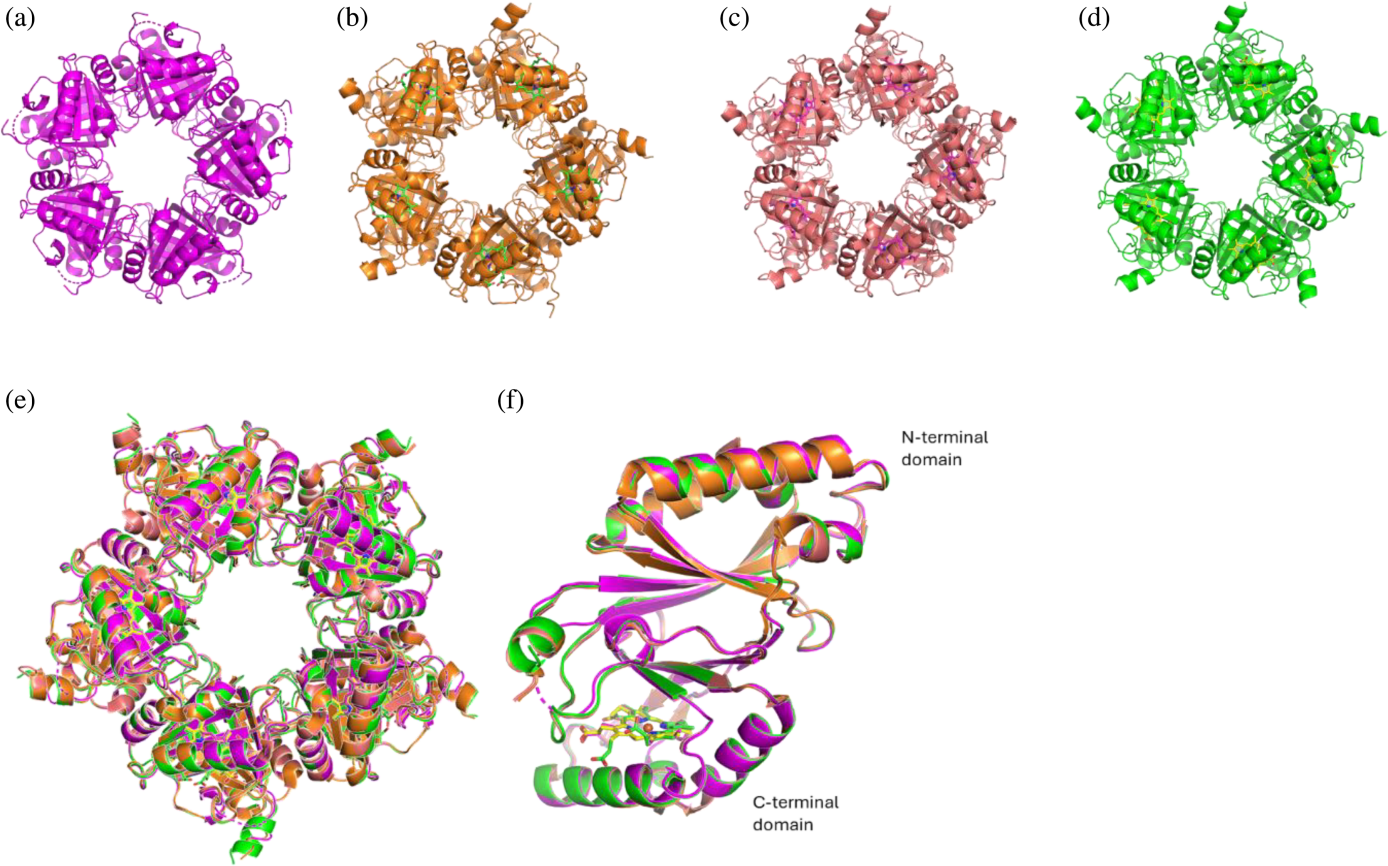
Comparison of overall structures of coproheme decarboxylase from *Corynebacterium diphtheriae* (*Cd*ChdC), upper panel. (a) Cryo‐EM structure of apo‐*Cd*ChdC protein, (b) crystal structure of coproheme‐bound *Cd*ChdC, and (c) MMD‐bound *Cd*ChdC, (d) Cryo‐EM structure of heme *b*‐bound *Cd*ChdC. Superimposition of experimental structures of *Cd*ChdCs, lower panel. (e) Cryo‐EM and X‐ray structures of apo‐*Cd*ChdCs (magenta, PDB ID: 8QWC), coproheme‐bound (orange, PDB ID: 6XUC), MMD‐bound (salmon, PDB ID: 6XUB), and heme *b‐*bound (green, PDB ID: 8QUO) were aligned such that the RMSD values of their domains are minimized. (f) Side view of superimposed single subunit of *Cd*ChdC.

An overall representation of all enzymatically relevant states (8QWC apo, 6XUC coproheme‐bound, 6XUB MMD‐bound, 8QUO heme *b*‐bound) of pentameric *Cd*ChdC is shown in Figure 2. When superimposed, both the cryo‐EM structures (PDB ID: 8QWC and 8QUO) were highly similar except for the heme pocket, with the average RMSD value of Cα displacement of 0.106 Å. Also, the RMSD value of Cα displacement between all the enzymatic states of *Cd*ChdC obtained from X‐ray crystallography and cryo‐EM showed a value of <0.5 Å, demonstrating that there are no major conformational changes in the structures (Figure 2e). The 3D structure of *Cd*ChdC is composed of two ferredoxin‐like folds, one is at the N‐terminus and the other one is at the C‐terminus, with the respective termini having an average B‐factor of 106.6 and 106.1 Å^2^ for apo protein and 81.3 and 77.3 Å^2^ for heme *b*‐bound protein (Figure 2f).

**FIGURE 3:**
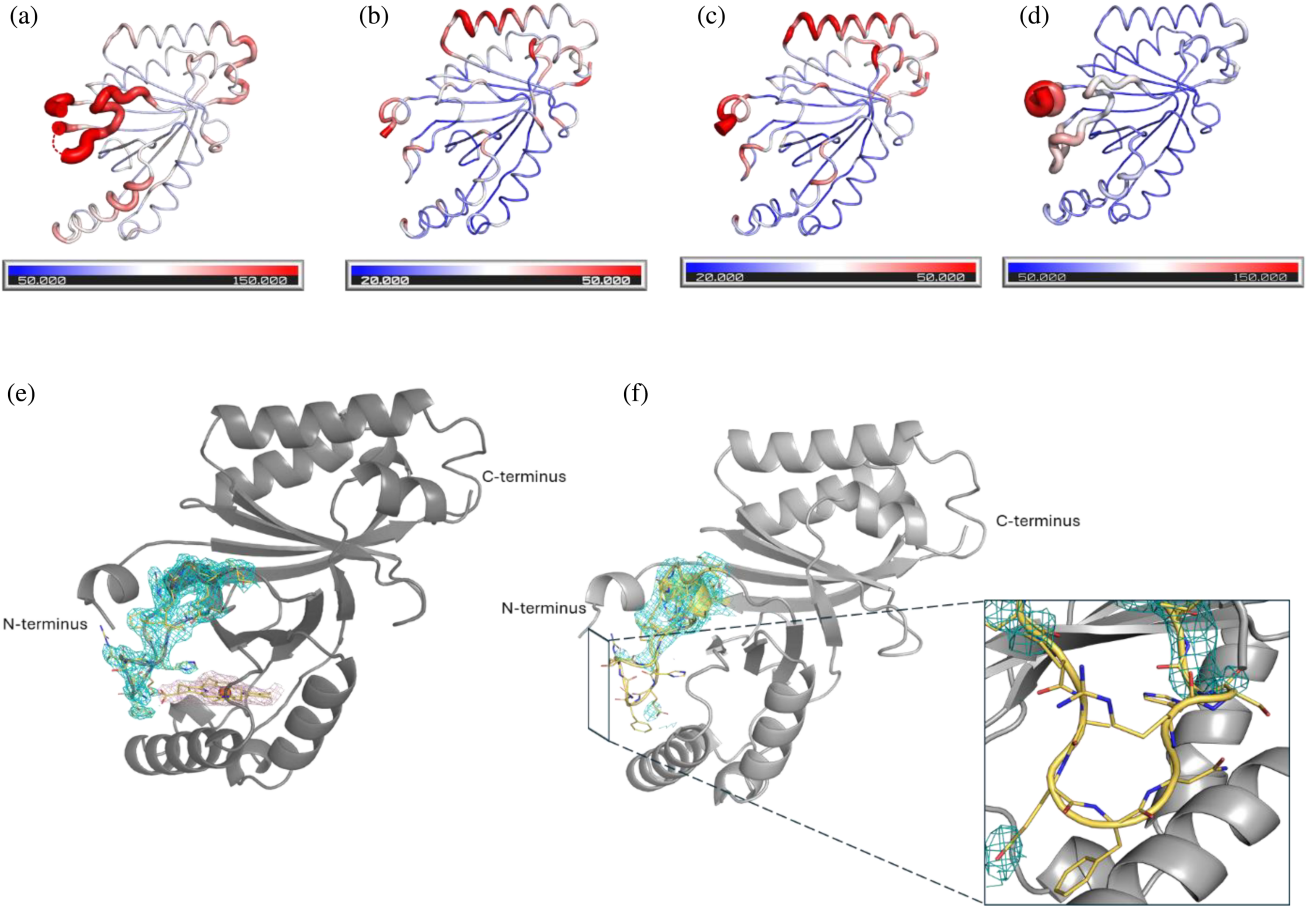
B‐factor variation in *Cd*ChdCs cryo‐EM and X‐ray structures (upper panel). B‐factor cartoon putty representation of (a) apo‐*Cd*ChdC, (b) coproheme‐bound, (c) MMD‐bound, and (d) heme *b*‐bound protomers. Loop resolution of cryo‐EM structures (lower panel). Side view of (e) heme *b*‐bound *Cd*ChdC. Heme *b* modeled into the cryo‐EM density in the active site with the map shown as magenta mesh and (f) apo‐*Cd*ChdC subunit with the loop region (L108–I127) represented in the molecular density map (blue mesh, the contour level is 8*σ*). The loop is shown as sticks with carbon, nitrogen, and oxygen colored yellow, blue, and red, respectively. Also labeled are the N‐termini and C‐termini.

The standard deviation (SD) of the average B‐factors of the C‐terminal domains in the X‐ray and cryo‐EM structures are larger than those of the N‐terminal domains. This is highly influenced by the loop region (L108–I127), which shows higher B‐factor in apo protein (145.6) followed by heme‐bound (99.9), MMD‐bound (71.0), and coproheme‐bound *Cd*ChdC (67.5) (Figure 3a–d). These observed differences in B‐factor values and the lack of electron density in the loop region of the *Cd*ChdC in the structure of the apo‐form is shown in Figure 3e,f.

### 3.2 Active site structure

**FIGURE 4:**
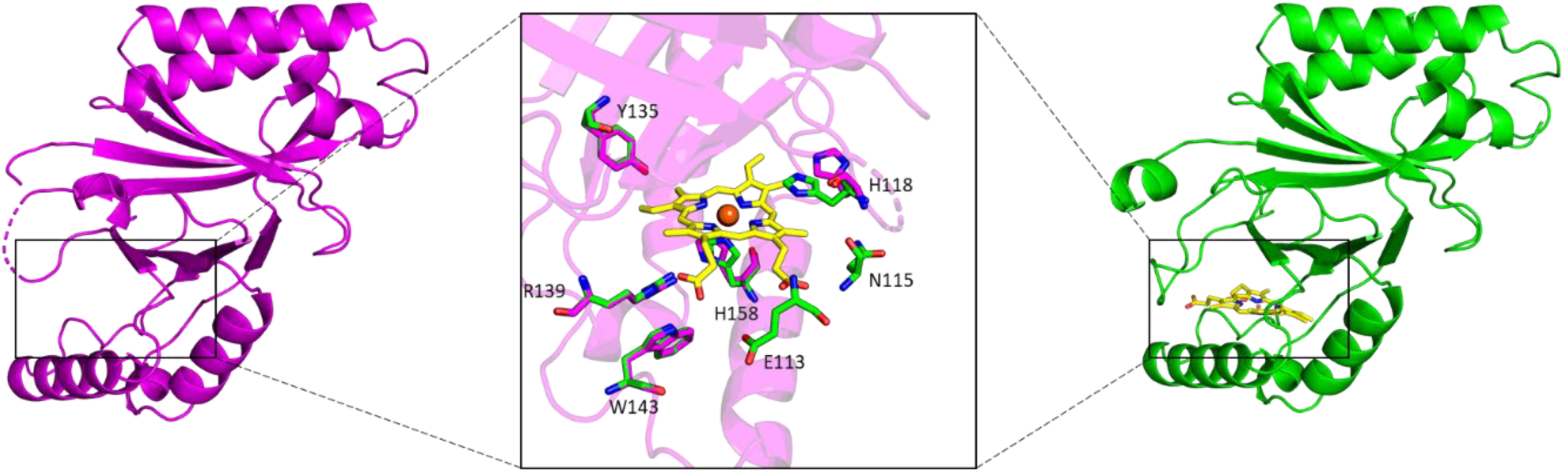
Active site architecture of *Cd*ChdCs. Superimposed active site residues of the cryo‐EM structure apo*Cd*ChdCs (magenta, PDB ID: 8QWC) and heme‐bound (green, PDB ID: 8QUO) represented using sticks. Heme *b* is shown in the center (yellow) with the two propionate groups.

The structures of all enzymatic states of *Cd*ChdC showed no major rearrangements, and the active site of apo‐*Cd*ChdC and heme *b‐*bound *Cd*ChdC in the cryo‐EM structure was also almost identical to that of coproheme‐bound and MMD‐bound *Cd*ChdC in X‐ray structures (Michlits et al. 2020). However, significant differences between the cryo‐EM and X‐ray structures of *Cd*ChdC have been observed for the loop between residues L108 and I127, at the access of the substrate binding site. In the 6XUC coproheme and the 6XUB MMD‐bound structures, the substrate is stabilized by multiple H‐bond interactions within the active site. These H‐bonds are formed between propionate 2 and 4 (in coproheme), and 4 and 6 (in MMD) and amino acid residues E113, N115, R139, W143, T205, R208. Similarly, the heme *b* in the active site of 8QUO is stabilized by hydrogen bonds formed by the two propionate groups p6 with R139 and W143, and p7 with E113 and N115 (Michlits et al. 2020; Milazzo et al. 2019b; Sebastiani et al. 2023). Also, the heme *b* formed after the second decarboxylative cleavage of propionate p2 does not show any further rotation in the active site, as it is bound in the same pose as MMD (Figure 4), which is evidenced by the positions of the remaining propionate groups 6 and 7. Such H‐bond interactions with the loop residues are absent in the cryo‐EM apo structure likely resulting in multiple conformations.

In principle, multiple distinct conformers of the loop can be resolved in cryo‐EM structures but dynamic disorder that we expect here averages out molecular density. As a result, in the 3D reconstructed map of the apo structure the loop density is only partially visible (Wu and Lander, 2020). The conformational change of the loop (L108–I127) in the apo structure seems to be accompanied by some additional conformational disorder of the N‐terminal residues (M1–L10) as they are located close to each other (Figure 3).

### 3.3 Molecular dynamics simulations

Molecular dynamics simulations were performed for apo‐*Cd*ChdC and heme *b‐*bound *Cd*ChdC mainly to determine the flexibility of the loop comprising residues 108–127. This includes comparing the loop residues involved in heme mobility and their role in stabilizing the protein. MD simulations of apo‐*Cd*ChdC showed noticeable relaxation from the starting geometry (PDB ID: 6XUB), which corresponds to the MMD‐bound protein. Structural features around the ligand‐binding groove were mostly affected, particularly the loop 108–127 and helix 140–160 (which includes the proximal histidine). These two structural features are of particular interest, as the loop 108–127 crosses over the ligand‐binding groove, conforming an obstacle to the ligand ingress/egress, and contains H118 which has been proved to act as a distal base for heterolytic cleavage of hydrogen peroxide (Michlits et al. 2020). Helix 140–160, on the other hand, contains the proximal histidine residue, which binds the iron (Fe) of the ligand. This residue plays a crucial role in the conformational variability of the heme‐like ligand, as well as residues R139 and W143 present on the loop binds to the propionate p6 in MMD (PDB ID: 6UXB) and heme *b*‐bound (8QUO) structures. Conformational variability in this region is of particular interest, as it may provide molecular insight into the detailed binding process of coproheme, unbinding of heme *b*, and the rotation of the porphyrin group in the proposed reaction mechanism. Propionate decarboxylation in actinobacterial coproheme decarboxylases follows a multi‐step mechanism, which is initiated by oxidation of coproheme to Compound I (oxoiron(IV) porphyryl radical) by deprotonated hydrogen peroxide (facilitated by H118). Compound I is converted by an internal electron transfer to Compound I* (oxoiron(IV) Y135^•^). Further, this neutral tyrosyl radical attacks the β‐carbon of the first propionate group at pyrrole ring A, resulting in release of carbon dioxide and formation of a vinyl group. The intermediate three‐propionate porphyrin undergoes a 90° reorientation and places the propionate of pyrrole ring B into the decarboxylation pocket. Cleaving of the second propionate is performed analogously to the first one (Michlits et al. 2020; Sebastiani et al. 2021).

**FIGURE 5:**
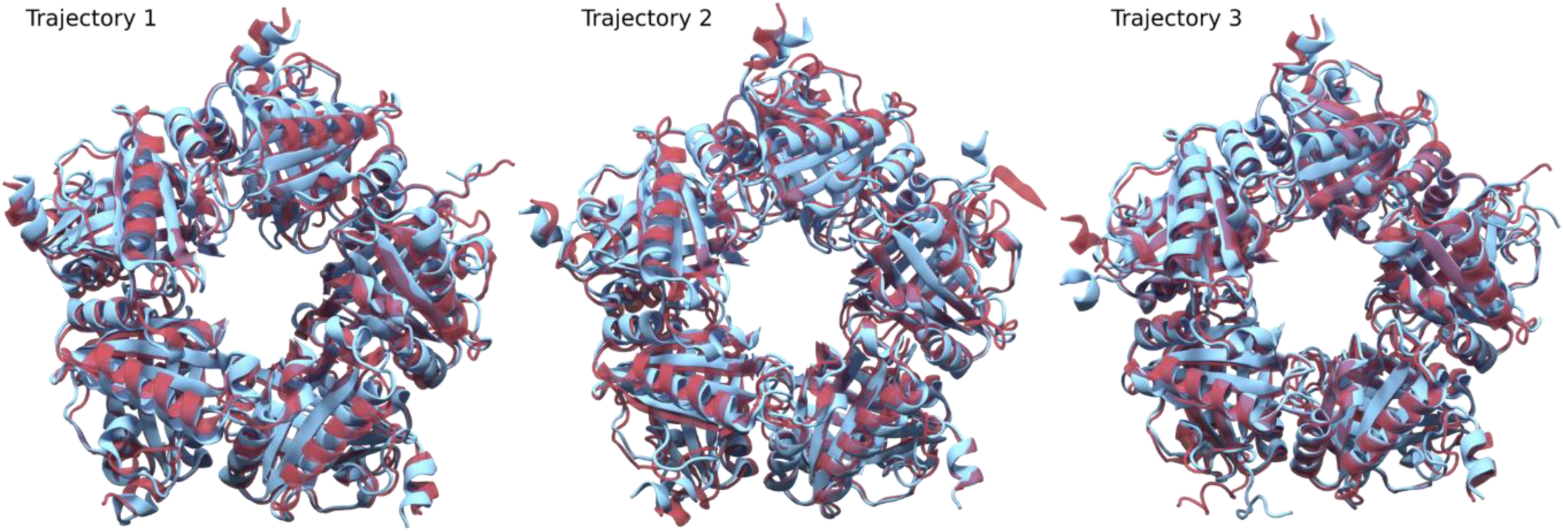
MD Simulations of *Corynebacterium diphtheriae* coproheme decarboxylase apo protein. Superposition of frames of an 800 ns trajectory (left panel), 600 ns (center panel), and 600 ns (right panel) of the apo‐*Cd*ChdC backbone in the cartoon representation. Red represents the starting frames while blue represents the end of the trajectory. Each frame is an average of 100 frames.

Figure 5 shows a comparison of initial and final frames (averaged over the first and last 100 ns of the trajectories) of three trajectories totaling 2 μs. Relaxation of previously mentioned structures is especially evident on the left panel. The N‐terminal region (adjacent to the 108–127 loop) also shows a very variable structure although this variability is seen across all simulations regardless of the presence or absence of ligand.

**FIGURE 6:**
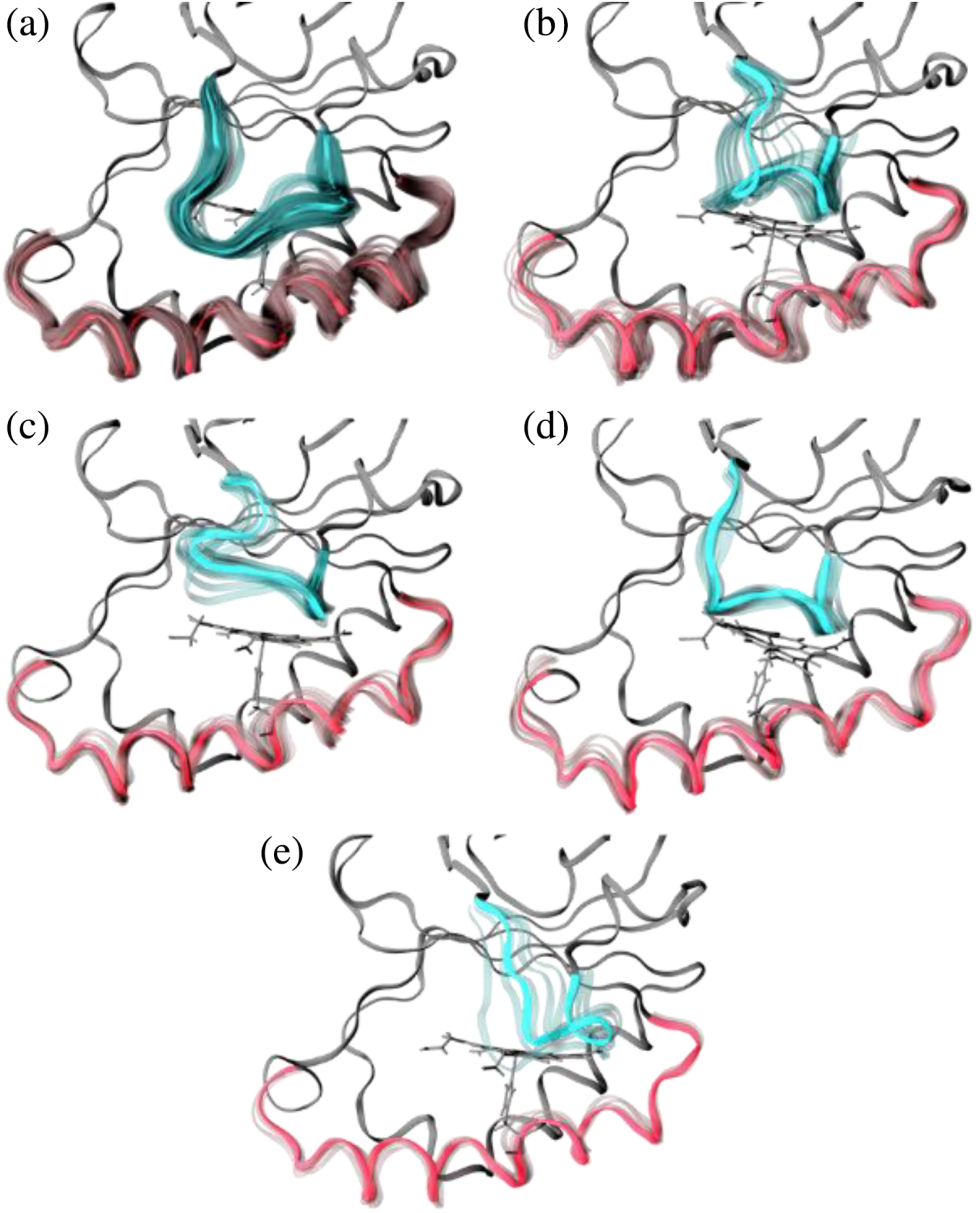
Representative structures of the five main clusters obtained from all heme b‐*Cd*ChdC complex trajectories pooled. Clusters 0 through 4 are shown in panels (a) through (e), respectively. Loop 108–127 highlighted in blue and helix 143–166 in red with the remaining backbone shown in gray. Alpha carbons of all protein subunits were fitted together to show all subunits superimposed. Average structures are shown in solid colors, while individual frames showing fluctuations are shown in transparency.

An agglomerative hierarchical clustering algorithm was used to form 15 clusters for the heme *b‐Cd*ChdC complex, of which its five most populated clusters accounted for 92.7% of the combined trajectory. The population percentages for clusters 0–4 were 57.4%, 12.7%, 9.1%, 9.0%, and 4.6%, respectively (Figure 6). The most populated cluster 0 is similar to the state found in crystalline structures 6UXC and 6UXB, representing what we call the “closed” form of the loop, followed by clusters 1 through 4 showing an “open” loop conformation.

**FIGURE 7:**
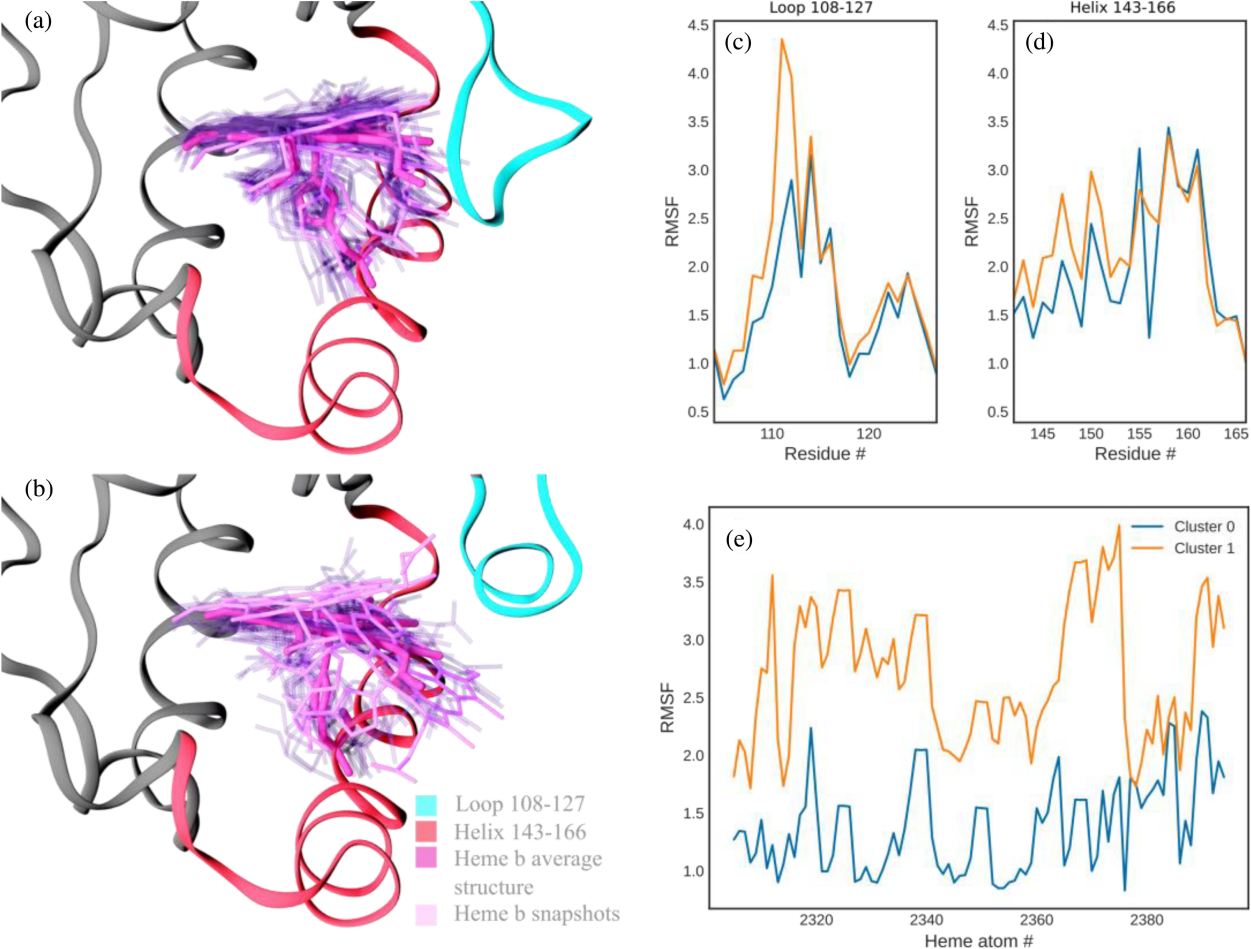
(a) Comparison of heme mobility for representative structures of cluster 0 (a) and 1 (b). Also, per residue RMSF results for loop 108–127 (c) and helix 143–166 (d), as well as per atom RMSF for the heme group (e). In (a, b) the protein backbone is depicted in ribbon representation. Loop 108–127 is shown in red, while helix 143–166 is shown in blue. Heme *b* group is shown in licorice representation in dark purple (average over the cluster), pink (highlighted frames), and transparent pink (frames taken every 100 representing fluctuations inside the cluster). The panels on the right show the RMSF data of cluster 0 in blue and cluster 1 in orange.

A comparative study of heme *b‐Cd*ChdC loop conformations from clusters 0 (closed conformation) and 1 (open conformation) are shown in the figure below (Figure 7a,b). Analyzing the phi and psi angles of residues in these clusters, we can confirm that in the open conformation, the loop takes on a more pronounced helical structure. Also, the heme group exhibits greater mobility in cluster 1 (open loop conformation) compared to cluster 0 (closed loop conformation), despite cluster 0 containing 53,000 frames while cluster 1 only contains 14,700 frames. Figure 7c,d displays the root‐mean‐square fluctuations (RMSF) per residue comparing both clusters for the loop as well as the helix regions. Figure 7e illustrates the RMSF per atom for the heme group, comparing clusters 0 and 1. Figure 7c,d shows greater mobility of the loop and helix regions in the open versus closed conformations (cluster 0 and 1, respectively). In Figure 7e the conformation of the loop is shown to have a strong effect on the heme *b* mobility as measured by its per atom RMSF. This implies a strong correlation between the heme group mobility and the loop 108–127 conformation in the heme *b* complex, with its mobility greatly increased for cluster 1 compared to cluster 0 as shown in Figure 7a,b.

**FIGURE 8:**
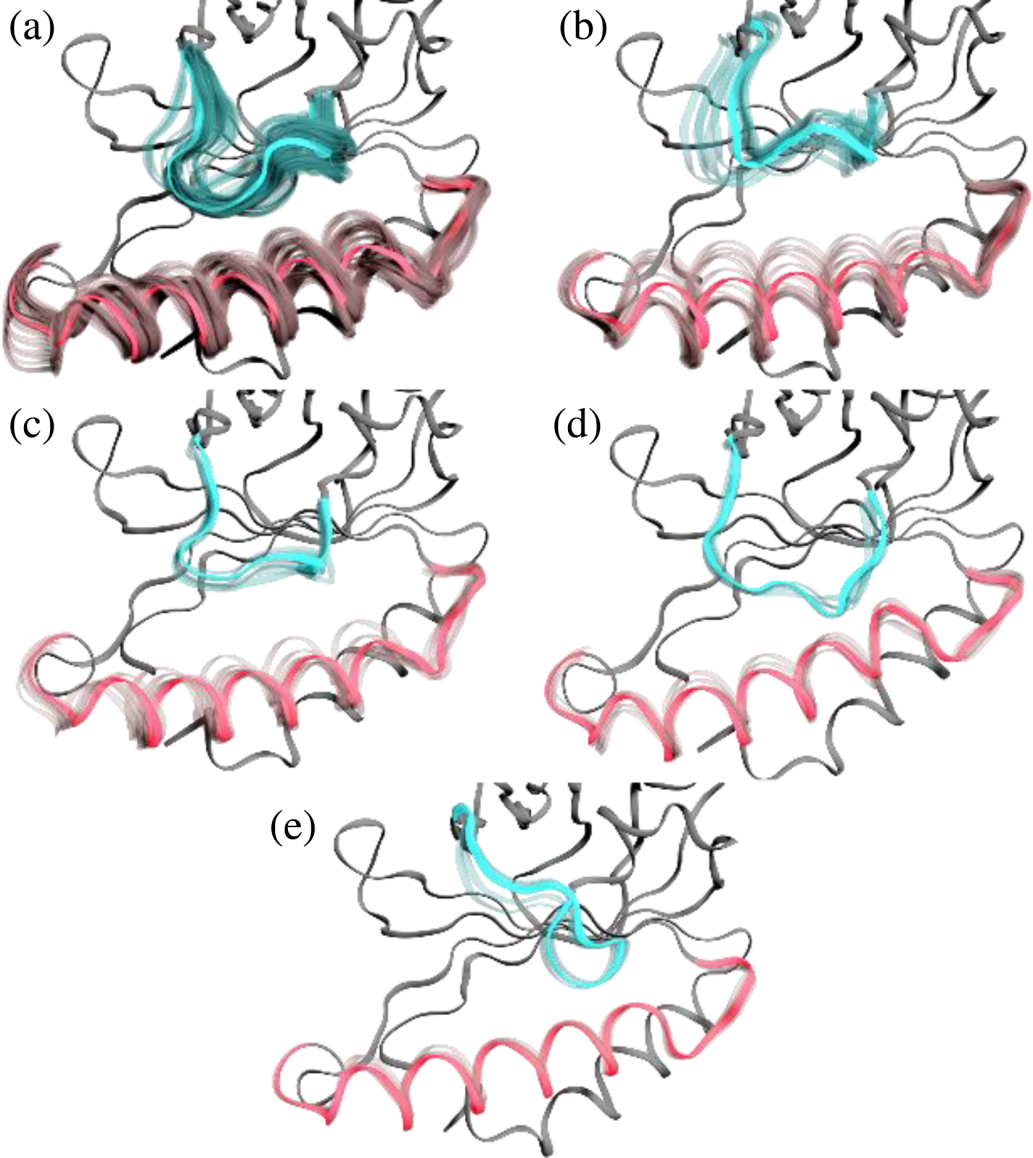
Representative structures of the five most populated clusters of apo‐*CdC*hdC. Clusters 0 through 4 are shown in panels (a) through (e), respectively. The backbone is shown in ribbons representation with loop 108–127 highlighted in blue and helix 143–166 in red with the rest of the backbone in gray. Alpha carbons of all protein subunits were fitted together to show all subunits superimposed. Average structures are shown in solid colors, while individual frames showing fluctuations are shown in transparency.

**FIGURE 9:**
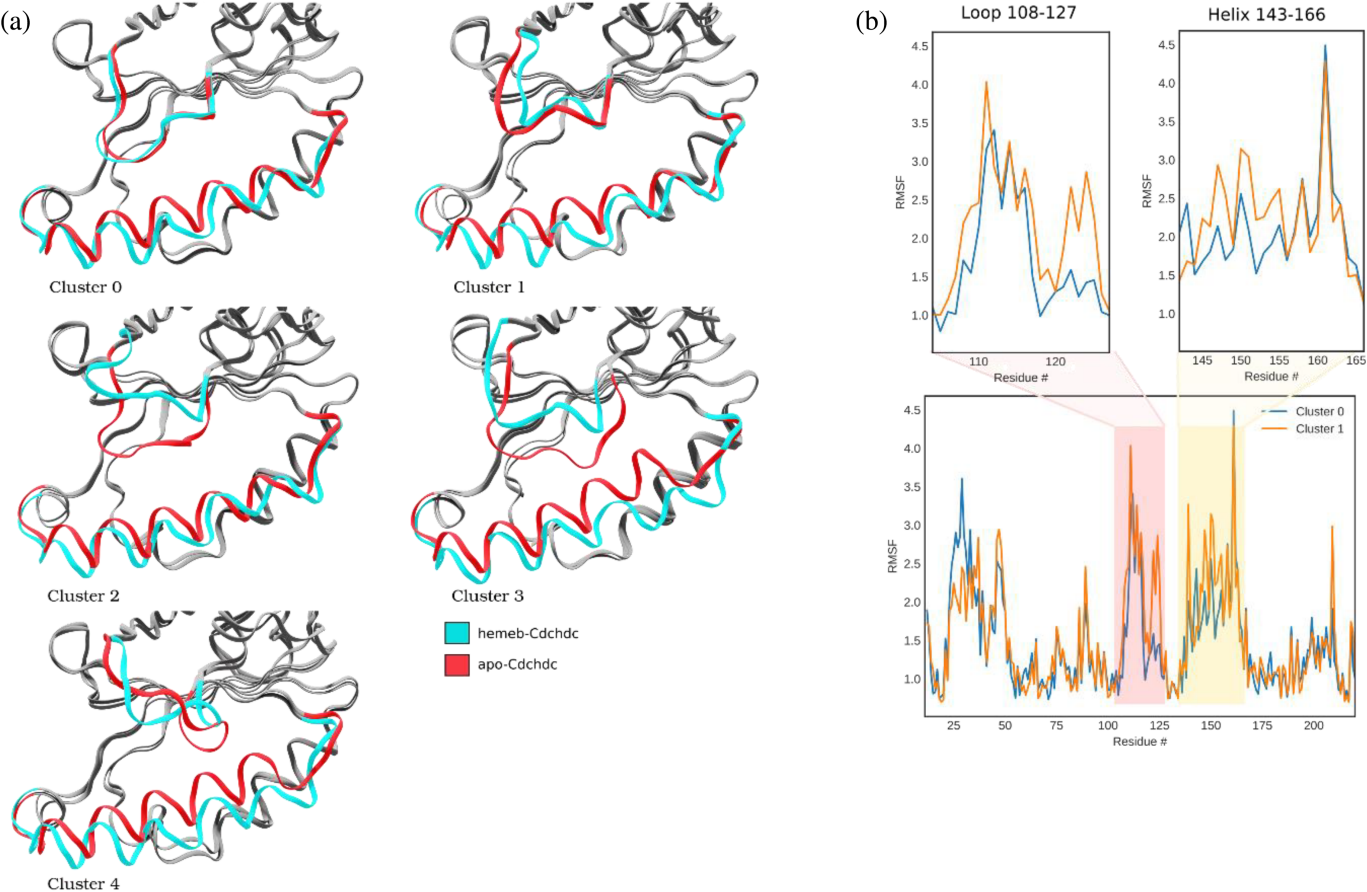
(a) Comparison of clusters obtained for apo‐*Cd*ChdC with those of the heme *b*‐*Cd*ChdC complex. (b) RMSF per residue for apo‐*Cd*ChdC for clusters 0 and 1. Clusters 0 through 4 are shown in panels (a) through (e), respectively. Ribbon representation is used. Loop 108–127 and helix 146–166 are shown in colors cyan and red for their respective structures, while the rest of the protein is shown in gray.

A similar clustering method was applied to the previously obtained apo‐*Cd*ChdC state trajectories. In this case, 15 clusters were obtained, with the first 5 clusters comprising 78.5% of the total trajectories (Figure 8). The population percentages for clusters 0–4 were 50.8%, 11.3%, 6.2%, 5.6%, and 4.6%, respectively. The apo form of the protein exhibits greater conformational variability, as indicated by the lower accumulated populations of the first five clusters (78.5% for apo vs. 92.7% of the heme *b* complex). When comparing the average structures for the first five clusters from the apo and the heme *b* complex, clusters 0, 1, and 4 are very similar (Figure 9a), while clusters 2 and 3 differ substantially. The clusters of the apo state also show the relaxation motion of helix 143–166 towards the interior of the binding groove. The high mobility of the loop in the apo state can also be observed when comparing the RMSF per residues of apo and heme *b* complexed proteins (Figure 9b).

The apo form shows increased RMSF of loops 108–127 in both clusters 0 and 1 compared to the heme *b* complex; however, the effect is more significant for cluster 1 (open loop) (Figure 9b). This implies that the loop has increased flexibility in the absence of heme *b*, which is consistent with our cryo‐EM data indicating a lack of loop density in the apo structure. The helix region 143–166 also shows slightly increased RMSF in the apo compared to the heme *b* complex. Also, a significant difference in the mobility of this helix region can be observed when comparing all five clusters from the apo state. The MD simulation demonstrates that the helix 140–160 is displaced towards the 108–127 loop, which aligns with our cryo‐EM data (see Figure 9a).

**FIGURE 10:**
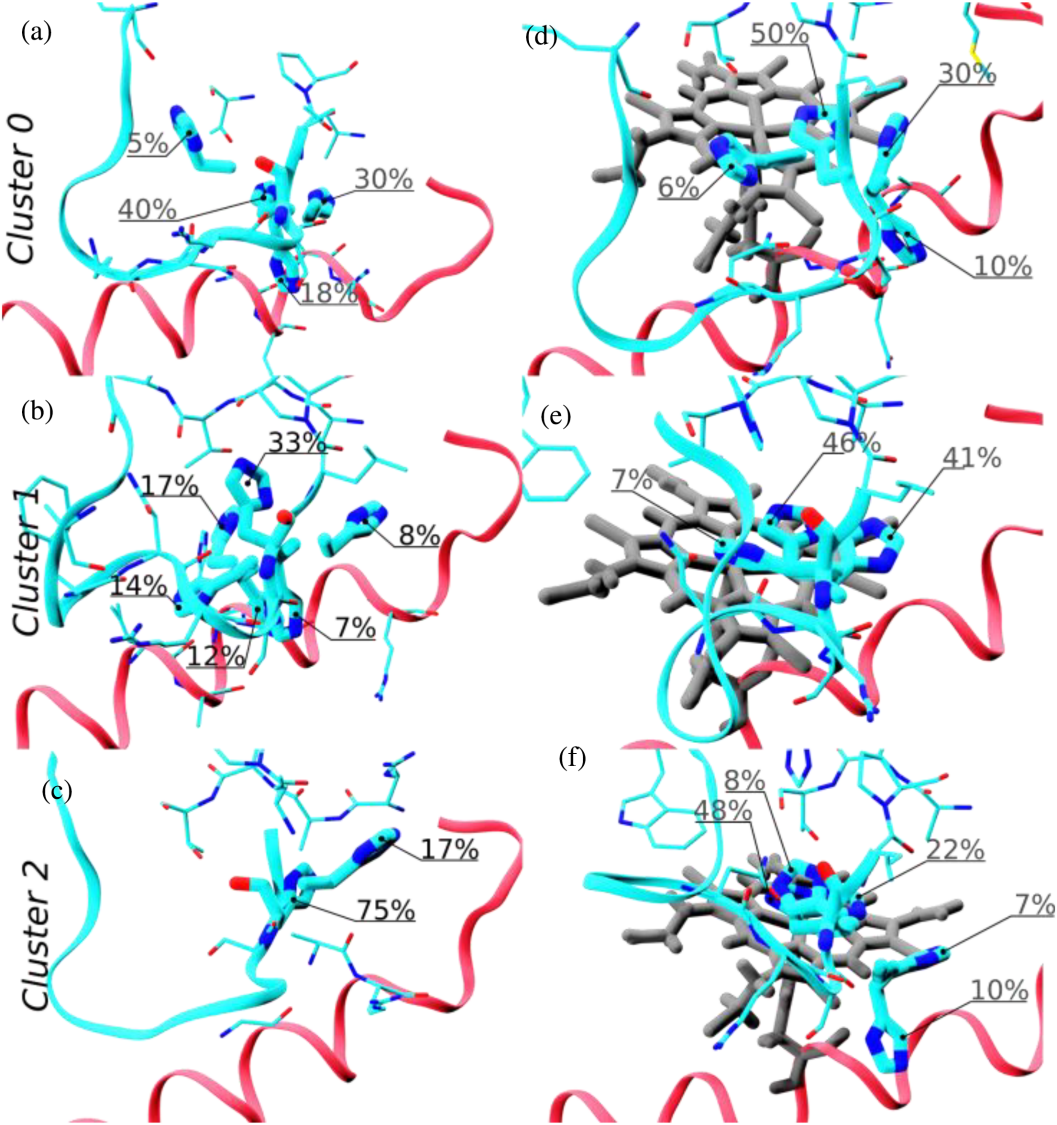
His118 conformations subclusters and populations distinguished by loop 108–127 conformation (clusterization analysis performed previously; see Figures 7, 8, 9, 10). The left panels correspond to apo‐*Cd*ChdC, while the right panels are heme *b*‐*Cd*ChdC. Loop 108–127 is shown as cyan ribbons and helix 140–160 in red ribbons. Heme is shown in gray. Residues close to His118 are shown as thin licorice and His118 as thick licorice. Populations of different His118 conformations are indicated as percentages of the total trajectory.

Figure 10 shows a clustering analysis of His118 conformations performed over the clusters previously found for the loop 108–127 for apo‐*Cd*ChdC (left panels) and heme‐*Cd*ChdC (right panels). In the case of heme‐*Cd*ChdC, all clusters show two main His118 conformations which align very well with the two alternative conformations found in cryo‐EM experiments (see Figure 4). The apo‐protein on the other hand shows less ordered His118 conformations. However, in the closed loop conformation (cluster 0) His118 shows very similar patterns in both proteins, with a greater preference for the histidine conformation hydrogen bonded to the Thr172 in heme‐*Cd*ChdC (50% for the heme‐*Cd*ChdC compared to 40% for apo‐*Cd*ChdC). Clusters 1 and 2 (open conformations of loop 108–127) for heme‐*Cd*ChdC show proportionally similar His118 conformations to cluster 0 which differ noticeably from those found in apo‐*Cd*ChdC.

**FIGURE 11:**
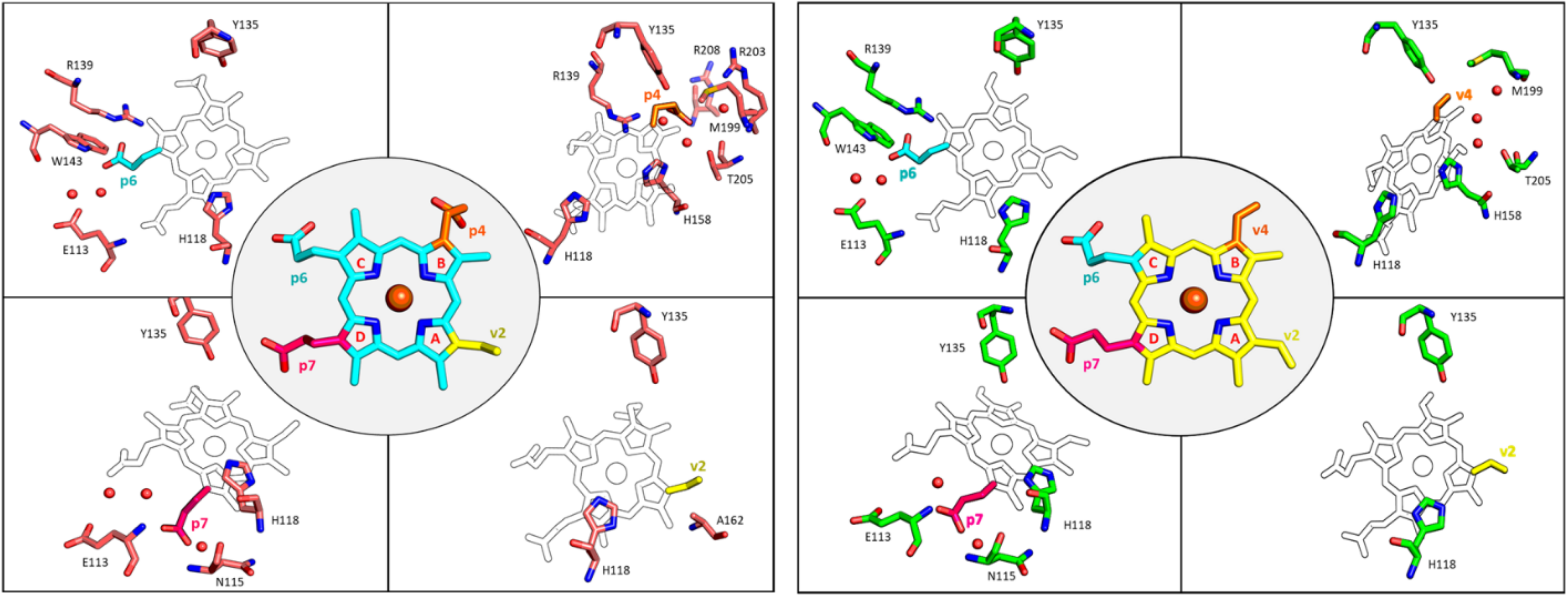
Porphyrin interactions. The hydrogen bond network of monovinyl monopropionate deteroheme (MMD) (left) and heme *b* (right) with their respective neighboring residues in the active site of *Cd*ChdC. The protein residues are depicted using the stick model, and the water is displayed as red spheres. A distinct color represents each propionate and its corresponding vinyl groups.

**FIGURE 12:**
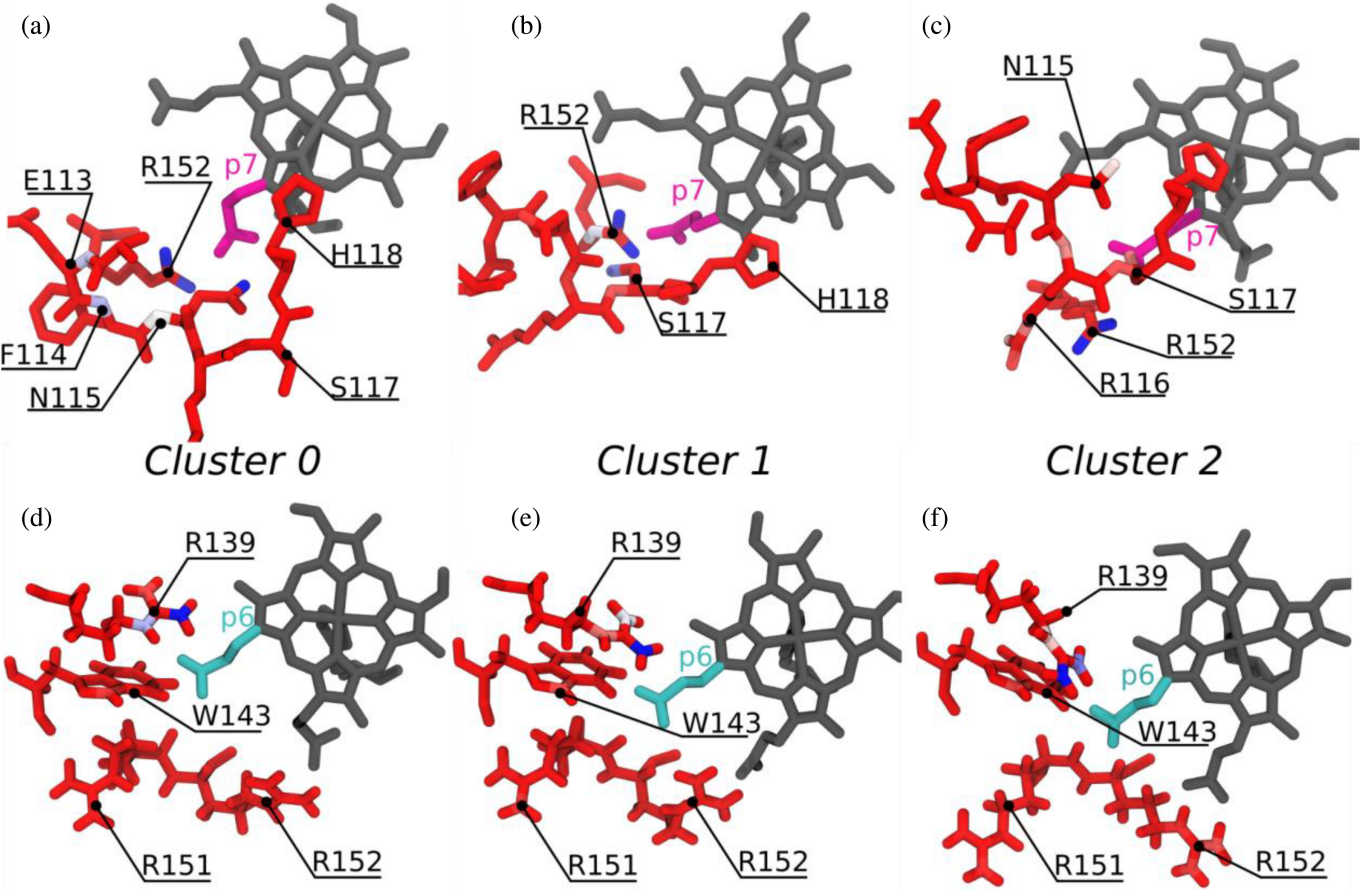
Contact maps of heme *b* propionates. Contact maps of propionate p7 (upper panels, a, b, and c) and p6 (lower panels, d, e, and f) with amino acid residues of heme‐*Cd*ChdC. Panels on the left (a, d) correspond to the loop 108–127 in the “closed” conformation (cluster 0), while those on the center (b, e) and right (c, f) correspond to the “open” conformation of loop 108–127 (clusters 1 and 2) (see Figure 6). Propionate p7 is shown in magenta in the upper panels, while p6 is shown in cyan in the lower panels. The color scheme of amino acid residues represents the probability of interaction with p6 or p7 at distances shorter than 3.5 Å, red is low, blue is high, and white is intermediate.

Figure 11 shows a hydrogen bond interaction map for monovinyl monopropionate deuteroheme (MMD), and heme *b* for the respective experimental structures; meanwhile, Figure 12 shows a contact map (for interactions shorter than 3.5 Å) for our simulations of heme‐*Cd*ChdC obtained for the different loop 108–127 clusters. Figure 12 shows that while interactions of propionate p6 remain largely the same and independent on loop 108–127 conformation, propionate p7 interaction changes significantly depending on if the loop 108–127 is in the “closed” (cluster 0) or “open” (clusters 1 and 2) conformations. Specifically, R152 shows a high level of interaction with p7 in all loop conformations, but E113, F114, and N115 show a high interaction probability (through their respective backbones) in the “closed” form (cluster 0), which disappear in the “open” loop conformations. In its place, heme propionate p7 interacts with the backbone of residues R116 and S117, although with a significantly lower probability. The side chains of residues N115 and S117 are worth a special mention. While N115 shows a strong interaction with p7 in the loop “closed” form, this interaction is entirely absent in cluster 1 and is severely weakened in cluster 2. Moreover, in the “open” loop conformations the side chain of residue S117 is moved to a more favorable position to interact with p7 in the “open” loop conformations, as shown in Figure 12b,c. It is therefore, possible to explain, at least in part, the greater conformational diversity of the heme group in the “open” loop 108–127 conformations with the weaker interactions observed between p7 and the loop amino acids.

## 4 DISCUSSION

The apo‐*Cd*ChdC cryo‐EM structure adds significantly to the structural knowledge on coproheme decarboxylases, it is also the first reported actinobacterial apo‐ChdC structure. It shows a complete absence of loop (108–127) densities for all five subunits. This contrasts with the other apo ChdC structures obtained through X‐ray crystallography (4WWS, 1VDH, 1T0T), which showed the loop densities present in some of the subunits, likely due to the influence of the crystal packing contacts (Ebihara et al. 2005; Hofbauer et al. 2015). Missing densities can also be observed for the N‐terminal regions. The loop connecting the two ferredoxin‐like domains within one subunit varies in sequence lengths between bacterial species. It adopts different conformations, modulating the shape of the substrate channels and the respective active site architectures, modifying substrate specificity, and optimizing enzymatic properties (Hofbauer et al. 2021).

Based on crystal structures and UV–vis stopped‐flow kinetics data, the previous studies indicate that catalytic tyrosine Y135 is crucial for catalysis in both Actinobacteria and Firmicutes (Celis et al. 2017; Milazzo et al. 2019a; Streit et al. 2018). Also, actinobacterial ChdCs use a conserved distal histidine residue (identified as H118 in *Cd*ChdC) as a base for deprotonation and heterolytic cleavage of incoming hydrogen peroxide. Unlike in Firmicutes, this catalytically important residue H118 (located 5 Å from the heme iron) in Actinobacteria is located on the loop connecting the N‐terminal and the C‐terminal ferredoxin‐like domains (Michlits et al. 2020). H118 establishes hydrogen bonds with propionate at position 6 (p6 of coproheme) and position 7 (p7 of MMD and heme *b*), contributing significantly to the molecular interactions at these specific sites in the resting state as well as during turnover. In the case of firmicutes *Listeria monocytogenes Lm*ChdC, the flexible loop contains a histidine (H117), firmly positioned at a distance of approximately 17 Å from the coproheme iron, as supported by its structural data (PDB ID: 6FXJ). Nevertheless, the role of H117 in *Lm*ChdC remains unequivocally unknown, but it seems not to play an essential role as a distal base, unlike in actinobacterial ChdCs (Milazzo et al. 2019a). The flexibility of the loop is stabilized by porphyrin binding in the active site. This structural study also indicates that histidine and other residues on the loop interact with the porphyrin structure of coproheme or heme *b*. These interactions are critical for stabilizing the loop, which contributes to the overall stability of the protein. The binding of coproheme or heme *b* induces specific conformational changes that reorganizes the loop, thereby preventing unwanted flexibility that could destabilize the active site. In heme *b‐Cd*ChdC the final product of the heme biosynthesis pathway is found in the active site of *Cd*ChdC and needs to be transferred to apo‐heme proteins to fulfill their physiological role. Therefore, heme *b* and the loop generate a metastable situation (Figure 7) that prevents heme *b* to be released into the cell, as free heme causes oxidative damage and therefore would be toxic, but renders the affinity low enough to pass heme *b* on to target proteins. Furthermore, these interactions play a role in the protein's function by correctly positioning the loop and its residues for catalysis or binding to other neighboring residues.

The position of heme *b* in the active site is stabilized by several H‐bond interactions between the remaining two propionate groups and the active site residues. Similar interactions are also present with the four‐propionate substrate coproheme and the three‐propionate intermediate MMD. The electron density at the active site in the MMD‐*Cd*ChdC structure (6XUB) and heme *b*‐bound structure exhibits minor changes, including the cleaved propionate p4 and the absence of interactions with surrounding residues. The heme *b*‐*Cd*ChdC structure reveals a distinct electron density of heme *b* in the active site of all monomers. The orientation is at a 90° angle compared to the active site of the coproheme‐bound structure (PDB ID: 6XUC), yet it aligns with the MMD‐bound structure, indicating no additional rotation of heme *b* in the active site. The absence of the p4 propionate group in the heme *b* structure eliminates the water‐mediated hydrogen bond with neighboring arginine residues to R203 and R208. Notably, no major rearrangements of active site residues are observed, which is crucial information for future possible drug design approaches. Even though the loop shows a high degree of flexibility, the rearrangement of the loop residues is not largely affected by coproheme, MMD, and heme *b*. The consistent active site configuration allows for precise computational modeling of drug binding. Thus, helping to build reliable pharmacophore models to facilitate high‐throughput in silico screenings of natural product databanks in the quest to find drug‐lead compounds. The stability and consistent positioning of these residues suggest that the active site maintains a rigid conformation upon porphyrin binding, providing a reliable target for the development of inhibitors. Moreover, the loop, with its higher degree of movement and conformational diversity compared to the active site residues, presents an attractive target for pharmacophore models. This flexibility allows for more diverse interactions and potential binding sites, which can be exploited in drug design to develop compounds that specifically modulate the loop's conformation and function. Understanding these dynamic interactions and structural stabilities can inform the design of drugs that precisely target these flexible regions, potentially leading to more effective therapeutic strategies.

## 5 CONCLUSION

In this work we complete the structural characterization of actinobacterial coproheme decarboxylase by presenting data on the two enzymatically relevant states that have not been described previously: the enzyme's apo‐form and the final product‐bound heme *b*‐bound form. The comprehensive structural characterization allows us to further investigate the active site architecture by molecular dynamics simulations to probe the behavior of the flexible loop (108–127) connecting the N‐terminal and the C‐terminal domains. This loop contains the catalytically important H118 and controls the access to the active site, where the substrate (coproheme) and the product (heme *b*) have to bind. Interactions of porphyrin propionate groups of coproheme and heme *b* with amino acid residues from *Cd*ChdC modulate the respective binding, which ultimately enables heme *b* to be released as the final product. Future detailed studies on the fate and on the trafficking of heme *b* to target proteins will be needed to further unravel heme homeostasis in Gram‐positive bacteria.

Structural data: PDB‐ID of apo‐*Cd*ChdC: 8QWC; PDB‐ID of heme *b*‐*Cd*ChdC: 8QUO.

## AUTHOR CONTRIBUTIONS

**Gaurav Patil:** Investigation; writing – original draft; methodology; validation; visualization; writing – review and editing; formal analysis; data curation. **Diego Javier Alonso de Armiño:** Data curation; formal analysis; software; visualization; validation; methodology; writing – review and editing; investigation. **Yirui Guo:** Supervision; formal analysis; software; data curation; writing – review and editing; methodology; resources; visualization. **Paul G. Furtmüller:** Writing – review and editing; resources; formal analysis. **Dominika Borek:** Methodology; writing – review and editing; software; formal analysis; supervision; data curation; resources; project administration. **Dario A. Estrin:** Conceptualization; supervision; resources; project administration; writing – review and editing; methodology. **Stefan Hofbauer:** Funding acquisition; conceptualization; investigation; writing – original draft; writing – review and editing; methodology; formal analysis; project administration; resources; supervision.

## CONFLICT OF INTEREST STATEMENT

Y.G. and D.B. are co‐founders of Ligo Analytics. Y.G. serves as the CEO of Ligo Analytics.

## Supporting information


**Figure S1.** Contact map for His118 in apo‐*Cd*ChdC MD simulations separated by loop 108–127 clusters. Cluster 0 (closed conformation) is panel (a). Panels (b), (c) and (d) are clusters 1 through 3 (open loop conformations). His118 is not shown. Cutoff for interactions is 7.0 Å. Blue represents a high interaction probability, red is low and white, intermediate.
**Figure S2**. Contact map for His118 in heme‐*Cd*ChdC MD simulations separated by loop 108–127 clusters. Cluster 0 (closed conformation) is panel (a). Panels (b), (c) and (d) are clusters 1 through 3 (open loop conformations). His118 is not shown. Cutoff for interactions is 7.0 Å. Blue represents a high interaction probability, red is low and white, intermediate.
**Figure S3**. Detail of loop 108–127 structure. Notice the differences in N115.
**Figure S4**. Histogram of the distance between NH2 of N115 sidechain and R110 backbone oxygen for cluster 0 (blue) and cluster 1 (red). There is a near perfect correlation between N115 hydrogen bonding R110 and the appearance of cluster 1 (open loop conformation).
