# Supplementary material for: Insights into the flexibility of the domain‐linking loop in actinobacterial coproheme decarboxylase through structures and molecular dynamics simulations
Source: Protein Sci. 2025 Jan 25;34(2):e70027. doi: 10.1002/pro.70027 (PMC11761711; doi:10.1002/pro.70027)
Supplement: Supplementary file 1 — Figure S1. Contact map for His118 in apo‐CdChdC MD simulations separated by loop 108–127 clusters. Cluster 0 (closed conformation) is panel (a). Panels (b), (c) and (d) are clusters 1 through 3 (open loop conformations). His118 is not shown. Cutoff for interactions is 7.0 Å. Blue represents a high interaction probability, red is low and white, intermediate. Figure S2. Contact map for His118 in heme‐CdChdC MD simulations separated by loop 108–127 clusters. Cluster 0 (closed conformation) is panel (a). Panels (b), (c) and (d) are clusters 1 through 3 (open loop conformations). His118 is not shown. Cutoff for interactions is 7.0 Å. Blue represents a high interaction probability, red is low and white, intermediate. Figure S3. Detail of loop 108–127 structure. Notice the differences in N115. Figure S4. Histogram of the distance between NH2 of N115 sidechain and R110 backbone oxygen for cluster 0 (blue) and cluster 1 (red). There is a near perfect correlation between N115 hydrogen bonding R110 and the appearance of cluster 1 (open loop conformation). [file PRO-34-e70027-s001.docx]

Supplementary Information

**Insights into the flexibility of the domain-linking loop in actinobacterial coproheme decarboxylase through structures and molecular dynamics simulations**

**Gaurav Patil^1^, Diego Javier Alonso de Armiño^2^, Yirui Guo^3,4^, Paul G. Furtmüller^1^, Dominika Borek^4,5^, Dario A. Estrin^2^, Stefan Hofbauer^1^***

^1^ BOKU University, Department of Chemistry, Institute of Biochemistry, Muthgasse 18, A-1190 Vienna, Austria

^2^ CONICET-Universidad de Buenos Aires, Instituto de Química Física de los Materiales, Medio Ambiente y Energía (INQUIMAE), Buenos Aires, Argentina

^3^ Ligo Analytics, Dallas, TX 75206, USA.

^4^ Department of Biophysics, The University of Texas Southwestern Medical Center, 5323 Harry Hines Blvd, Dallas, TX 75390, USA.

^5^ Department of Biochemistry, The University of Texas Southwestern Medical Center, 5323 Harry Hines Blvd, Dallas, TX 75390, USA.


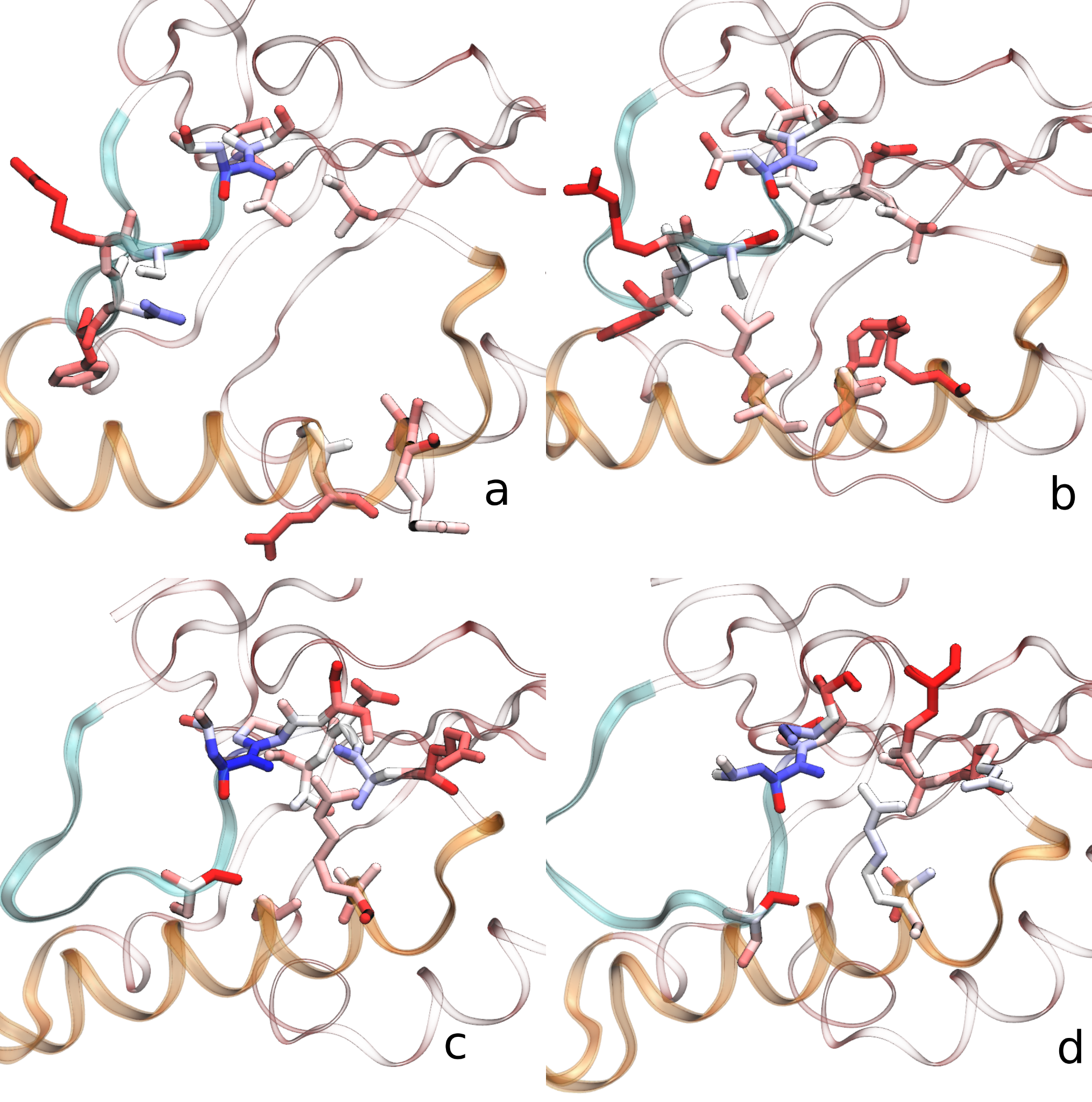


Figure S1. Contact map for His118 in apo-*Cd*ChdC MD simulations separated by loop 108-127 clusters. Cluster 0 (closed conformation) is panel (a). Panels (b), (c) and (d) are clusters 1 through 3 (open loop conformations). His118 is not shown. Cutoff for interactions is 7.0 Å. Blue represents a high interaction probability, red is low and white, intermediate.


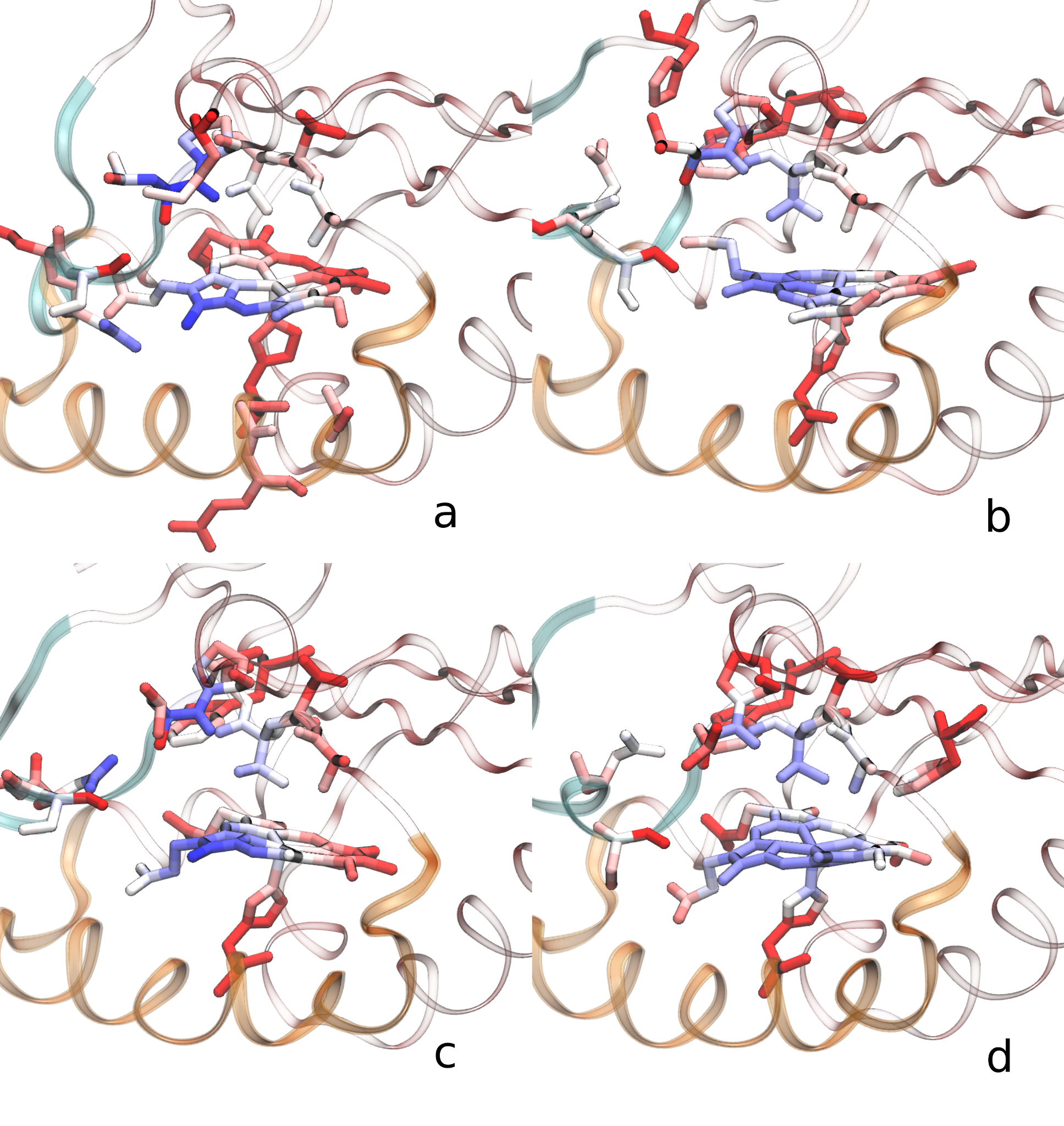


Figure S2. Contact map for His118 in heme-*Cd*ChdC MD simulations separated by loop 108-127 clusters. Cluster 0 (closed conformation) is panel (a). Panels (b), (c) and (d) are clusters 1 through 3 (open loop conformations). His118 is not shown. Cutoff for interactions is 7.0 Å. Blue represents a high interaction probability, red is low and white, intermediate.


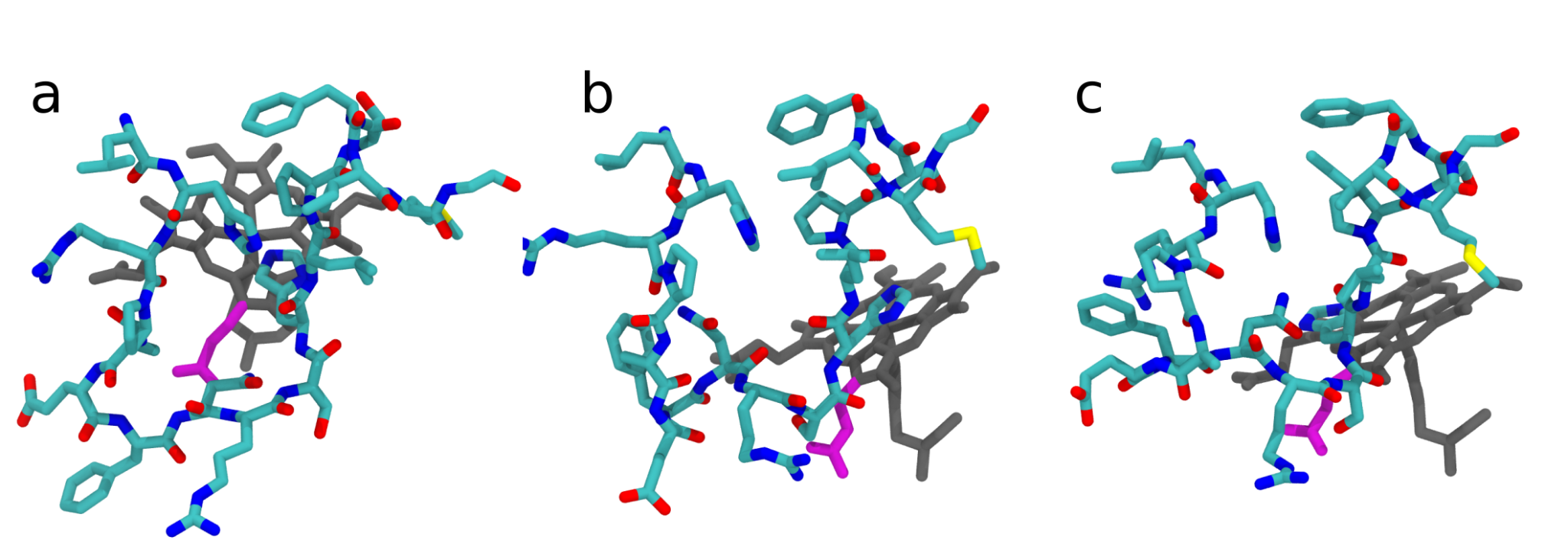


Figure S3. Detail of loop 108-127 structure. Notice the differences in N115.


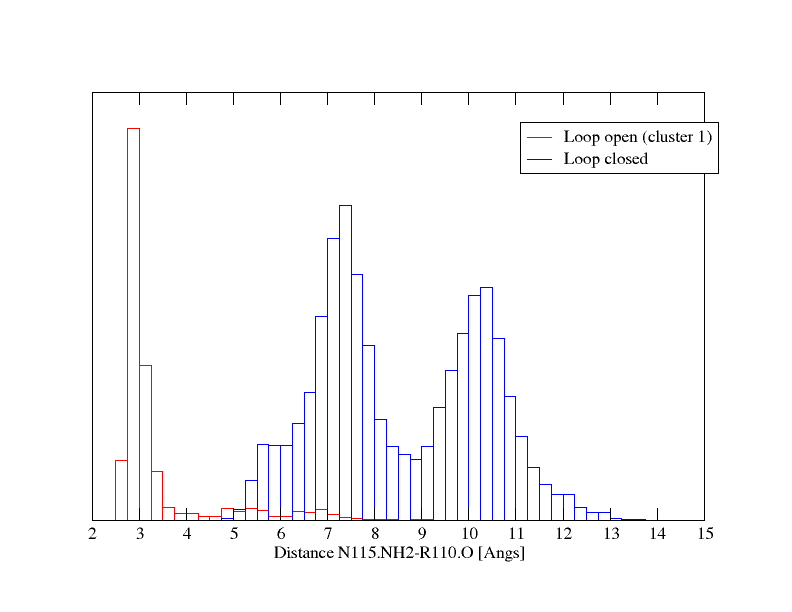


Figure S4. Histogram of the distance between NH2 of N115 sidechain and R110 backbone oxygen for cluster 0 (blue) and cluster 1 (red). There is a near perfect correlation between N115 hydrogen bonding R110 and the appearance of cluster 1 (open loop conformation).
